# Unveiling Selected Influences on Chronic Kidney Disease Development and Progression

**DOI:** 10.3390/cells13090751

**Published:** 2024-04-26

**Authors:** Piotr Fularski, Witold Czarnik, Hanna Frankenstein, Magdalena Gąsior, Ewelina Młynarska, Jacek Rysz, Beata Franczyk

**Affiliations:** 1 Department of Nephrocardiology, Medical University of Lodz, ul. Zeromskiego 113, 90-549 Lodz, Polandmagdalena.gasior@stud.umed.lodz.pl (M.G.);; 2 Department of Nephrology, Hypertension and Family Medicine, University of Lodz, ul. Zeromskiego 113, 90-549 Lodz, Poland

**Keywords:** chronic kidney disease, renin–angiotensin–aldosterone system, TGF-β1, vascular calcification, uremic toxins, hypertension

## Abstract

Currently, more and more people are suffering from chronic kidney disease (CKD). It is estimated that CKD affects over 10% of the population worldwide. This is a significant issue, as the kidneys largely contribute to maintaining homeostasis by, among other things, regulating blood pressure, the pH of blood, and the water–electrolyte balance and by eliminating unnecessary metabolic waste products from blood. What is more, this disease does not show any specific symptoms at the beginning. The development of CKD is predisposed by certain conditions, such as diabetes mellitus or hypertension. However, these disorders are not the only factors promoting the onset and progression of CKD. The primary purpose of this review is to examine renin–angiotensin–aldosterone system (RAAS) activity, transforming growth factor-β1 (TGF-β1), vascular calcification (VC), uremic toxins, and hypertension in the context of their impact on the occurrence and the course of CKD. We firmly believe that a deeper comprehension of the cellular and molecular mechanisms underlying CKD can lead to an enhanced understanding of the disease. In the future, this may result in the development of medications targeting specific mechanisms involved in the decline of kidney function. Our paper unveils the selected processes responsible for the deterioration of renal filtration abilities.

## 1. Introduction

The kidneys are a highly significant component in terms of maintaining the homeostasis of the human body. They are responsible for various functions, including, among others, preserving acid–base, water, and ion balance; eliminating waste products of metabolism; and secreting specific hormones such as erythropoietin, angiotensin, and aldosterone or selected enzymes like renin. What is more, they are also capable of regulating blood pressure (BP) [1]. Unfortunately, the number of patients with kidney function disorders, in the form of chronic kidney disease (CKD), is increasing from year to year. It is estimated that this disease affects between 11.7% and 15.1% of the global population [2]. CKD is diagnosed based on a sustained decrease in kidney function lasting at least 3 months. This is understood as a decline in the glomerular filtration rate (GFR) below 60 mL/min per 1.73 m^2^ or by the presence of kidney damage markers such as hematuria or albuminuria. The term CKD also encompasses structural changes in the kidneys persisting for at least 3 months in imaging examinations, as takes place, for instance, in autosomal dominant polycystic kidney disease [3]. CKD also includes abnormalities in histological examination or kidney transplantation in the patient’s history. However, the disease does not present any specific symptoms at the beginning, which makes screening and awareness of factors that may lead to its development crucial, especially when it comes down to setting a proper diagnosis [4]. Particular attention should be paid to patients suffering from arterial hypertension, diabetes mellitus, autoimmune disorders, persistent pyelonephritis, and persistent glomerulonephritis and in individuals chronically taking non-steroidal anti-inflammatory medications [5]. In an era where dietary supplements are commonly consumed by patients, it is important to also exclude aristolochic nephropathy, which may occur as a result of taking so-called Chinese herbs [6]. CKD is a serious condition; however, it can be divided into certain categories allowing for the determination of disease advancement. It is described by category G, based on the GFR, and category A, based on the level of chronic albuminuria, which can be showed by the albumin excretion rate or the albumin–creatinine ratio in urine. The respective stages along with their corresponding values are presented in Table 1 and Table 2 [7].

As CKD progresses, there is a decrease in the kidney’s filtration capacity, expressed through the GFR [8]. Along with the decrease in the GFR, there is an accumulation of so-called uremic toxins, which do contribute to further disease progression. However, this is not the only mechanism leading to the aggregation of factors that ultimately worsen kidney function [9]. What is more, focusing solely on uremic toxins can be insufficient in terms of inhibiting the progression of CKD. There are also other elements contributing to the progression of this disease, such as vascular calcification (VC), which not only leads to further loss of kidney function but also promotes atherosclerosis development. Arterial hypertension’s contribution to the advancement of CKD must also be duly acknowledged [10,11]. CKD also signifies the presence of an inflammatory process within the kidneys, which further leads to the secretion of an increased amount of pro-fibrotic agents, such as transforming growth factor-β1 (TGF-β1). This aggravates the course of the disease by causing renal fibrosis and can further progress towards end-stage renal disease (ESRD) [12]. Moreover, increased activity in the renin–angiotensin–aldosterone system (RAAS) also advances the disease exacerbation [13]. The mentioned determinants such as uremic toxins, VC, hypertension, TGF-β1, and RAAS activity constitute components of CKD progression, to which we aim paying particular attention in this review.

## 2. Factors Contributing to the Deterioration of Renal Function and Progression of CKD

### 2.1. Renin–Angiotensin–Aldosterone System (RAAS) in Chronic Kidney Disease

**Figure 1:**
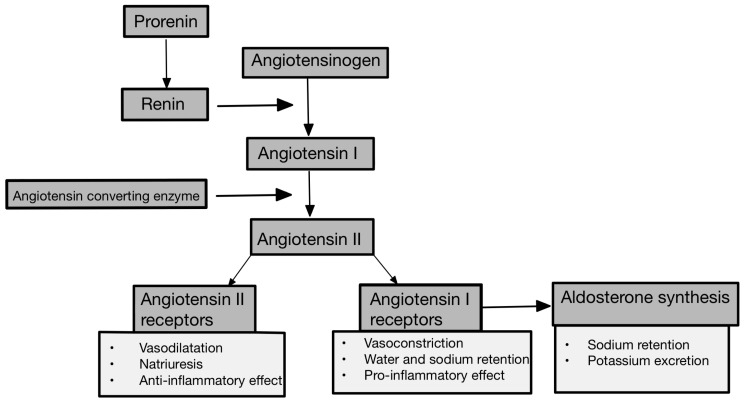
Physiological activation of RAAS and its selected clinical implications [17,20].

Already in the developing kidney, the RAAS components are significantly expressed and exert a crucial influence on shaping renal morphology and ensuring proper physiological function in later life [14]. In studies of renal programming, researchers have found that disruptions in the RAAS early in life can lead to long-term consequences. Physiologically, there is a temporary decline in infancy, followed by a normalization of RAAS components. However, certain factors can interfere with this process, leading to excessive RAAS activation in adulthood, raising BP and increasing the risk of kidney disease. In addition, abnormal RAAS activation can extend across generations, as observed in models involving a maternal high-fructose diet. Understanding these transgenerational effects may be critical to understanding the broader implications for kidney health [14,15]. Renin, angiotensin II (Ang II), and aldosterone are the three main components of the RAA system, which affect BP and body fluid homeostasis. Prorenin, stored by cells called juxtaglomerular cells, is secreted in an inactive form and converted to renin via the pro-renin receptor by the cleavage of a 43-amino acid N-terminal fragment after juxtaglomerular cell activation [14,16]. Juxtaglomerular cells are located in the walls of renal afferent arterioles at the entrance of the glomerular capillary network. Beta-activation and prostaglandins stimulate renin synthesis and secretion, in contrast to high BP, hypernatremia, volume overload, and Ang II, which inhibit secretion [17]. Once renin has been released into the blood, it can act on angiotensinogen—the target of renin [16]. Renin is responsible for the process of converting angiotensinogen to angiotensin I (Ang I). Angiotensinogen is mainly synthesized and released into the bloodstream from the liver, e.g., in response to inflammation, insulin, and estrogen. It is worth noting that organs like the heart and kidneys also have the capability to produce angiotensinogen [18]. Ang I promotes the secretion of epinephrine and norepinephrine [19]. In the next step, Ang I is converted to Ang II by angiotensin-converting enzyme 1 or 2 (ACE1/ACE2). ACE is primarily known for its ability to cleave Ang I to Ang II, while it cleaves not only Ang I but also many other substrates, including bradykinin [14]. Ang II is the main effector peptide of the RAAS and affects two types of receptors, angiotensin II type 1 (AT1) and angiotensin II type 2 (AT2). In classical pathway stimulation, AT1 receptors provide vasoconstriction, water, and sodium retention, aldosterone synthesis, and pro-inflammatory effects and promote growth and remodeling. AT2 receptors act oppositely to AT1 receptors, which means vasodilatation and nitric oxide (NO) production [19,20]. The level of Ang II is higher in the kidney than in any other tissue in the body, reflecting its key role in maintaining kidney function. Long-term Ang II stimulation in the kidney can lead to renal tubular acidosis; the increased production of reactive oxygen species; mitochondrial dysfunction; mesangial contraction; myofibroblast accumulation or tubular hypertrophy; and atrophy, atherosclerosis, ventricular hypertrophy, or myocardial infarction [19,21]. One of the major effects of Ang II is also the stimulation of aldosterone synthase by the adrenal cortex [22]. We can also distinguish angiotensin III (Ang III) and angiotensin IV (Ang IV), which are derivatives of Ang II, formed by the actions of enzymes like ACE1, aminopeptidase A, and aminopeptidase N. While both Ang III and Ang IV interact with AT1 receptors, they exhibit distinct preferences for other receptors. Ang III, known for its vasoconstrictive properties, shows higher affinity for AT2 receptors. On the other hand, Ang IV primarily targets angiotensin type IV (AT4) receptors, which are widely distributed in organs like the brain, lungs, and kidneys, where they play roles in cognitive and proliferative functions [23]. The alternative pathway of the RAAS involves the formation of angiotensin 1–7 (Ang 1–7) and angiotensin 1–9 (Ang 1–9) from Ang I and II via enzymes such as ACE2. Ang 1–7 binds to Mas receptors (MasR), counteracting the effects of the classic RAAS by promoting vasodilation, anti-inflammatory prostaglandin synthesis, and NO release, thereby lowering BP [24]. It also benefits the heart by reducing cardiomyocyte proliferation and hypertrophy and promotes coronary vessel regeneration and remodeling. Additionally, it regulates sodium transport, enhances glucose reabsorption, and protects against insulin resistance [25]. Ang 1–9, derived from Ang I, acts similarly to Ang 1–7 but has a stronger effect on heart tissue. It acts through AT2 receptors and can be converted directly into Ang 1–7 by specific enzymes [25,26]. A recent addition to the RAAS family, known as alamandine or Ala1-Ang-(1–7), has been discovered as a derivative of Ang 1–7 through decarboxylation. Alamandine can also be produced from Ang II through decarboxylation to form angiotensin A, which is then converted to alamandine by the enzyme ACE2. This peptide demonstrates antihypertensive and anti-fibrotic effects by binding to the Mas-related G-protein-coupled receptor, member D (MrgD) [27]. Aldosterone has a mineralocorticoid activity and is a key regulator of sodium, potassium, and body fluid balance. Physiologically, it is mainly responsible for sodium reabsorption in the distal nephrons. Is classified as a steroid hormone that binds the cytosolic mineralocorticoid receptor in the distal convoluted tubule of the kidney [28]. CKD has been linked to elevated aldosterone levels in the blood. High aldosterone levels correlate with scarring of renal tissue, increased renal vascular resistance, and proteinuria [29]. Unfortunately, it also promotes cardiac hypertrophy and fibrosis and induces podocyte injury and oxidative stress in vascular smooth muscle cells, especially in the presence of a diet high in salt [28]. Therefore, chronic activation of the RAAS promotes cardiovascular disorders such as congestive heart failure syndromes, systemic hypertension, and urinary system disorders, especially CKD [30]. Thus, excessive stimulation of the RAAS leads to the development of chronic heart failure (CHF). CHF causes a decrease in circulating blood volume. It also means that blood flow in the renal arteries is reduced, leading to the development of chronic renal failure. Also, contraction of the glomerular afferent arterioles aggravates renal ischemia and therefore reduces the GFR [19]. The progression of CKD accelerates to increase Ang II and aldosterone synthase, hypertension, or chronic inflammation. The physiological activation pathway of the RAA system and its selected clinical implications are shown in Figure 1.

### 2.2. Impact of TGF-β on Chronic Kidney Disease

A multifunctional cytokine, namely TGF-β, belongs to the transforming growth factor superfamily that includes three different mammalian isoforms (TGF-β 1 to 3), HUGO Gene Nomenclature Committee (HGNC) symbols (TGF-β1, TGF-β2, and TGF-β3), and many other signaling proteins that are significant to mammalian development and homeostasis [31]. TGF-β is a highly pleiotropic cytokine that plays a major role in wound healing, angiogenesis, immunoregulation, and cancer [32]. In the pathogenesis of CKD, TGF-β1 plays a crucial role, since long-term TGF-β1 overproduction can lead to end-stage diabetic nephropathy (DN) [33]. It also plays an important role in kidney fibrosis development and inflammation through its downstream signaling cascades, which activate cellular pathologic mechanisms underlying the progression of renal inflammation and fibrosis. Nephritis often leads to the initiation of renal fibrosis. In acute and chronic renal injury, cytokine release, inflammatory cell infiltration, and the subsequent epithelial-to-mesenchymal transition lead to fibrosis and renal failure [34]. Mechanisms of tubular repair include the activation of the epithelial growth factor receptor (EGFR). Acute activation of the EGFR is beneficial in the early stages of renal injury; however, chronic activation leads to renal fibrosis [35,36,37]. EGFR activation increases TGF-β1 expression, resulting in the stimulation of interstitial myofibroblast proliferation. This induces the secretion of collagen and other extracellular matrix proteins and leads to interstitial fibrosis and the functional failure of nephrons [38]. In renal tissue, CKD is linked to significant alterations in cell signaling, such as the activation of TGF-β1, p53, and developmental genes such as *Wnt* and *Notch* [39]. p53 is a well-known tumor suppressor protein that is a key component of the cellular response to stress. The regulation of p53 includes its expression and stability in concert with a wide variety of reversible post-translational modifications (PTMs). In addition to its well-known function as a tumor suppressor, p53 has a much broader role, and its dysregulation contributes to the development of a variety of human diseases. p53 is involved in the development of acute kidney injury (AKI) and subsequent renal repair mainly through the regulation of apoptosis, cell cycle arrest, and autophagy. Sustained arrest of the tubular cell cycle in the G2/M phase results in abnormal repair and subsequent progression to CKD after AKI [40]. Considering etiology, heightened TGF-β levels and the TGF-β1/Smad3 signaling pathway are highly correlated with the promotion of the transcription of several pro-fibrotic genes and drive fibroblast activation, which leads to disease progression [41]. TGF-β1 plays a critical role in the initiation and advancement of renal fibrosis; its effector Smad proteins have diverse and even antagonistic roles in fibrosis regulation [31]. TGF-β1 directly activates Smad signaling, which triggers the overexpression of pro-fibrotic genes. Numerous studies have shown that dysregulation of the TGF-β1/Smad pathway is an important pathogenetic mechanism in tissue fibrosis. Smad2 and Smad3 are the two main regulators that promote TGF-β1-mediated tissue fibrosis, while Smad7 serves as a negative feedback regulator of the TGF-β1/Smad pathway and thereby protects against TGF-β1-mediated fibrosis [42]. The precise mechanisms that mediate TGF-β1-induced fibrosis, however, are not well understood [43].

### 2.3. Vascular Calcification in Chronic Kidney Disease

CKD patients display a very high percentage of vascular calcification (VC) [44], leading to cardiovascular disease, decreased life expectancy, and mortality [45] even in the earliest phases of CKD. In CKD patients, we observed significant disturbances in serum phosphorus, calcium, and the calcium–phosphorus product, which are implicated in the promotion of VC [46]. Even at early ages, CKD patients develop VC in almost all localizations in a greater proportion than the general population. This is frequently localized in high-caliber arteries, such as the aorta (79%) and medium arteries (70.5%), including coronary arteries, and also in small-caliber arteries (20.2%) [47]. Moreover, the calcification on the intimal and medial layer is more frequent in patients with CKD in comparison with the general population. Recent evidence has suggested that CKD is an aging-related disease that synergizes “pro-aging” molecules aggravating VC [48]. Parathyroidal hormone (PTH), vitamin D3, and other metabolites become dysregulated, causing mineral and bone metabolism disorders, termed CKD-mineral and bone disorders (CKD-MBDs), in CKD progression. CKD-MBDs significantly accelerate VC processes. The tremendous prevalence of cardiovascular morbidity is due to CKD-related metabolism disorders, and typical disorders include hypocalcemia, hyperphosphatemia, and secondary hyperparathyroidism [49]. Hyperphosphatemia is probably caused by decreased renal tubular excretion, subsequently stimulating PTH secretion and accelerating bone turnover and calcium deposition in arterial walls [50]. The dysregulated metabolites circulate in the vascular system and induce adverse effects on vascular smooth vessel cells, initiating inflammation, oxidative stress, and VC processes. Studies have indicated that elevated phosphates and PTH upregulate the expression of leucine-rich repeat-containing G-protein-coupled receptor 4, thus activating the parathyroid hormone dependent on the protein kinase A PTH/PKA signal pathway [51].

### 2.4. Uremic Toxins

**Figure 2:**
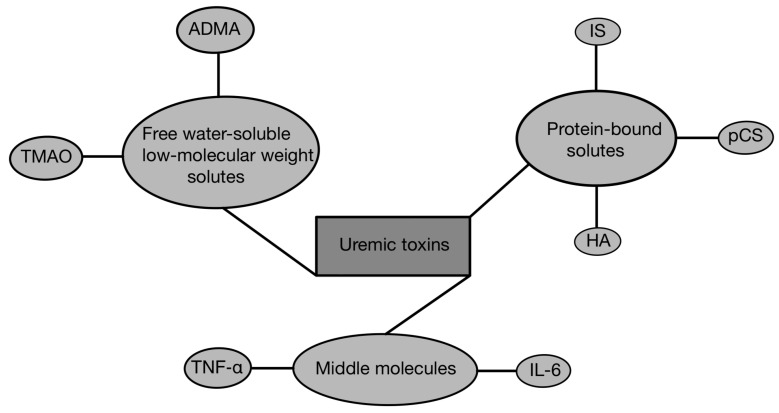
Classification of uremic toxins based on solubility [10]. ADMA—asymmetric dimethylarginine; TMAO—trimethylamine N-oxide; TNF-α—tumor necrosis factor α; IL-6—interleukin 6; HA—hippuric acid; pCS—p-cresyl sulfate; IS—indoxyl sulfate.

The kidneys play a crucial role in removing metabolic waste products from the body. In patients with CKD, toxins that are typically eliminated from the body under physiological conditions accumulate, disrupting the body’s homeostasis and deteriorating the patient’s quality of life. The contemporary understanding of uremic toxicity encompasses a variety of solutes that accumulate in the body as a result of kidney failure, exhibiting diverse physicochemical characteristics and causing various adverse effects on biological systems [52]. Uremic toxins are generally categorized into three groups: protein-bound solutes, free water-soluble low-molecular-weight solutes, and middle molecules, as presented in Figure 2. The accumulation of uremic toxins in the human body contributes to the progression of CKD [10].

#### 2.4.1. Protein-Bound Solutes

Indoxyl sulfate (IS) is a low-molecular-weight toxin classified among protein-bound solutes, with approximately 93% of its binding affinity attributed to albumins [53]. It is the end product of tryptophan supplied to the body through dietary intake. Tryptophan enters the intestines, where it is metabolized into Indole by gut bacteria through bacterial tryptophanase [54]. Indole then reaches the liver via portal circulation and is further metabolized into IS [55]. The elimination of IS from the body occurs through secretion in the proximal renal tubules via organic anion transporter 1 (OAT1) and organic anion transporter 3 (OAT3) [56]. Due to its significant association with serum proteins, IS is removed from the body to a small extent via dialysis (less than 35%) [57]. With the deterioration of kidney function in CKD patients, the concentration of IS in the body increases, leading to the progression of CKD through various mechanisms, such as inducing fibrosis and inflammation, exerting nephrotoxic effects by generating reactive oxygen species, and depleting antioxidative systems. IS stimulates the epithelial–mesenchymal transition (EMT) of renal tubular epithelial cells, considered a crucial mechanism contributing to fibrosis. EMT stimulation occurs through both TGF-independent and TGF-dependent mechanisms [58]. Additionally, IS activates tubulointerstitial inflammation by inducing the expression of intercellular adhesion molecule 1 (ICAM-1) and monocyte chemoattractant protein (MCP-1), which is associated with the infiltration of monocytes and macrophages into the kidney [59]. IS stimulates NF-kB, leading to the activation of p53, which in turn induces the expression of TGF-B1 and PAI-1, thereby promoting fibrosis-related processes [60]. Klotho is an anti-aging gene known for its renoprotective properties. In vitro and in vivo studies demonstrate that IS induces the depletion of Klotho, correlating with an elevated activation of NF-kB [61]. The depletion of Klotho induced by IS may potentially be facilitated epigenetically through CpG hypermethylation of the Klotho gene [62].

Another molecule within the group of protein-bound solutes is p-cresylsulfate (pCS). Intestinal bacteria metabolize phenylalanine and tyrosine, leading to the formation of phenolic compounds such as p-cresol [63]. Subsequently, p-cresol enters the liver, where it is sulfated by SULT1A1, giving rise to pCS [64]. pCS is excreted into urine through OATs, especially OAT3 located in the basolateral membrane, and among patients with CKD, there is an increase in its concentration in the serum. Similar to IS, pCS exhibits a high degree of binding with albumins, and only a small fraction is eliminated from the body through dialysis (less than 35%) [57]. The accumulation of pCS in the body, similar to IS, instigates renal fibrosis and inflammation by activating the RAAS, intensifying oxidative stress and promoting hypermethylation of the renoprotective Klotho gene [63]. Furthermore, pCS has been observed to impede the activity of efflux transporters such as multidrug resistance protein 4 (MRP4) and breast cancer resistance protein (BCRP) within proximal tubule cells. This inhibition has the potential to result in the intracellular accumulation of various toxins [65].

Hippuric acid is a protein-bound solute uremic toxin with the least scientifically explored impact on the progression of CKD. Intestinal bacteria metabolize polyphenolic compounds obtained from the diet into benzoic acid, which subsequently undergoes transformation into hippuric acid through conjugation with glycine in the kidneys or liver. Hippuric acid is bound to albumins to the extent of 34%, and in dialyzed patients, the clearance of hippuric acid during dialysis amounts to 64%. The elevation of hippuric acid concentration in the bodies of patients with CKD induces oxidative stress, thereby promoting endothelial dysfunction and renal fibrosis [66].

#### 2.4.2. Middle Molecules

The group of middle molecules includes pro-inflammatory cytokines such as tumornecrosis factor alpha (TNF-α) and interleukin 6 (IL-6). TNF-α is produced by immune system cells such as mast cells, macrophages, and lymphocytes, as well as by cardiomyocytes, vascular endothelial cells, mesangial cells, and tubular epithelial cells of the kidney [67]. The primary contributors to IL-6 synthesis are macrophages and monocytes. Furthermore, TNF-α, along with other pro-inflammatory cytokines like interleukin 1α (IL-1α), enhances the production of IL-6. During the course of CKD, the concentration of these pro-inflammatory cytokines significantly increases in the plasma [68].

TNF-α leads to a reduction in the GFR by decreasing the bioavailability of nitric oxide (NO) through increased superoxide generation and hemodynamic alterations resulting from the binding to tumor necrosis factor receptor type 1 (TNFR-1) [69]. Additionally, the stimulation of tumor necrosis factor receptor type 2 (TNFR2) leads to macrophage infiltration into the renal interstitium, contributing to glomerulosclerosis and interstitial fibrosis [70]. The main function of IL-6 is to regulate adaptive and innate immune responses. IL-6 signaling occurs through two pathways: in the classical pathway, IL-6 binds to the membrane-bound IL-6 receptor (mIL-6R), which leads to activation of the signal transducer glycoprotein 130 (gp130). On the other hand, the trans-signaling pathway occurs through the presence of soluble IL-6R (sIL-6R) in body fluids. It is believed that the classical pathway of IL-6 activation is responsible for anti-inflammatory effects and inhibits fibrotic processes in nervous tissue, whereas the pro-inflammatory properties of IL-6 are propagated through trans-signaling, leading to fibrosis in nervous tissue, as demonstrated in mouse models [71].

#### 2.4.3. Free Water-Soluble Low-Molecular Weight Solutes

Trimethylamine N-oxide (TMAO) is a pro-inflammatory free water-soluble solute synthesized in the liver from the precursor protein trimethylamine (TMA) through enzymatic reactions catalyzed by monooxygenases like FMO1 and FMO3 [72]. Gut bacteria, especially Firmicutes and Proteobacteria, play a role in generating TMA from choline, L-carnitine, and betaine, which are supplied through food. Foods particularly rich in TMAO precursors include red meat, fish, and eggs [73,74]. The elimination of TMAO occurs through the kidneys. Studies have shown that serum TMAO levels in individuals with impaired renal function are significantly higher than in healthy populations [75]. The efficacy of removing TMAO from plasma through a single hemodialysis is approximately 70% [76]. TMAO is a protein primarily associated with coronary atherosclerosis [77], but there is also evidence suggesting that the accumulation of TMAO may contribute to the progression of CKD. This occurs due to TMAO inducing the upregulation of pro-fibrotic genes and fostering tubulointerstitial fibrosis. Studies conducted on mice models have demonstrated that dietary supplementation of choline and TMAO leads to an increase in TMAO levels in the serum and contributes to elevated collagen deposition, tubulointerstitial fibrosis, and the phosphorylation of Smad3, which acts as a promoter of fibrotic processes in renal tissue [78]. Another free water-soluble uremic toxin is the non-proteinogenic amino acid asymmetric dimethylarginine (ADMA), which is derived from arginine through post-transcriptional methylation carried out by protein arginine methyl-transferases (PRMTs) [79]. The efficiency of plasma clearance of ADMA through a singular hemodialysis session is estimated to be approximately 23% [80]. In vitro studies have demonstrated that ADMA leads to the upregulation of NAD(P)H oxidase 4 (NOX4), resulting in the generation of reactive oxygen species (ROS). Consequently, this leads to the phosphorylation of extracellular-signal-regulated kinase (ERK1/2) and the activation of myofibroblasts [81]. The impact of different molecules on CKD is presented in Table 3.

## 3. Hypertension and Chronic Kidney Disease

Hypertension and CKD are tightly interconnected conditions, where persistent high BP can exacerbate kidney function decline, and conversely, worsening kidney function can impair the control of BP [82]. Hypertension represents the primary controllable risk element for heart disease, stroke, and kidney dysfunction [83]. Consistent BP measurement is crucial for diagnosing and effectively managing hypertension in CKD. We define hypertension as BP above 140/90 mmHg. Essential hypertension is more common in industrialized countries and is associated with risk factors such as aging, obesity, insulin resistance, diabetes, and hyperlipidemia [84]. Resistant hypertension is identified when patients have uncontrolled BP despite taking three or more antihypertensive drugs at adequate doses, or when patients are on four or more antihypertensive drug classes regardless of BP control, provided that antihypertensive treatment includes diuretics [85,86]. The prevalence of resistant hypertension is reported to be 2–3 times higher in patients with CKD than in the general hypertensive population [87]. Hypertension is one of the main risk factors for the development of CKD and progression to end-stage renal failure; it also increases in parallel with worsening stages of CKD [88,89]. Uncontrolled hypertension in patients with CKD increases the risk of cardiovascular events, one of the major causes of death in patients with CKD [89]. BP is predominantly controlled through four pathways, encompassing sodium regulation, activity in the sympathetic nervous system (SNS), the RAAS, and autoregulatory mechanisms. These pathways may exert independent or interconnected influences on BP regulation. Pathological changes in one or multiple factors, combined with additional external factors, can impact BP and its control in individuals with CKD [83]. The rise in sympathetic activity, triggered by signals from deteriorating kidneys, plays a role in causing high BP in CKD. As kidney function worsens, there is an increase in the activity of the RAAS, leading to greater retention of salt and water [90,91]. The first mechanism of sodium retention in the body is the influence of the sympathetic nervous system. Increased activity of sympathetic nerves in the kidneys leads to the retention of sodium by triggering the secretion of renin, reducing renal blood flow, and enhancing the reabsorption of sodium in the renal tubules [92]. Studies conducted in animals subjected to high-salt diets and norepinephrine infusion revealed that the reduction in urinary sodium excretion induced by norepinephrine is caused by two mechanisms. Firstly, it involves the stimulation of α-adrenergic receptors, which decreases renal flow and enhances tubular sodium reabsorption. Secondly, it entails the activation of β-adrenergic receptors, which in turn activates the thiazide-sensitive sodium chloride cotransporter (NCC). These observations indicate that heightened levels of sympathetic nerve activity (SNA) in the kidneys trigger the activation of the NCC, leading to the retention of sodium and the development of salt-sensitive hypertension [93,94]. Increased SNA is also associated with obesity. It has been demonstrated that leptin released by adipocytes stimulates the increase in SNA, which may fuel the mechanism development of obesity-related hypertension through sodium retention and also shows correlation between a high-fat diet and hypertension [95,96]. The effect of salt intake on BP differs between individuals. Patients with essential hypertension can be divided into salt-sensitive and salt-resistant groups. The second mechanism leading to increased sodium retention in the body is related to activity of the epithelial Na+ channel (ENaC), which plays a critical role in sodium homeostasis and the pathogenesis of salt-sensitive hypertension [97]. In salt-sensitive phenotype rats, a high-sodium diet has been shown to increase the renal GTPaseRac1, resulting in increased mineralocorticoid receptor activity, followed by increased sodium reabsorption in the collecting ducts through activation of the ENaC and the development of salt-sensitive hypertension [98]. There is also a potential connection between inflammatory factors, ENaC activation, and salt-sensitive hypertension [97]. It was discovered that sodium-induced activation of the NLRP3 inflammasome is ENaC and isolevuglandins (IsoLG) dependent. NLRP3-deficient mice develop a blunted hypertensive response to elevated sodium, and this is restored by the adoptive transfer of NLRP3 replete APCs [99]. The association between obesity and hypertension is extensively documented. CKD prevalence is higher among individuals with obesity and metabolic syndrome [83]. Obesity and the associated inflammation also plays an important role in ENaC activation. Individuals with both obesity and hypertension display heightened levels of plasma aldosterone, and these levels are strongly linked to BP in obese individuals [100,101]. Hypertensive rats with obesity exhibit hyperaldosteronism due to the release of aldosterone from the adrenal glands, which is stimulated by factors released by fat cells, such as tumor necrosis factor (TNF), complement C1q tumor necrosis factor-related protein 1 (CTRP1), interleukin-6 (IL-6), and oxidative by-products of linoleic acid. Despite inhibition of the RAAS, plasma aldosterone levels remain inadequately reduced by a high-salt diet in obese hypertensive rats, leading to increased mineralocorticoid receptor activity, increased ENaC activity, and elevated BP and damage to the heart and kidneys [102,103]. The issue of arterial hypertension is therefore very complex and depends on many factors. It has also been proven that advanced kidney disease is characterized by endothelial dysfunction, which is associated with the development of hypertension [104]. Other pathophysiological mechanisms of hypertension are an increased intracellular calcium level or the reversal of hypoxia-induced vasodilation [83]. The problem of nocturnal hypertension in CKD patients should also be mentioned, as it is a stronger predictor of cardiovascular complications and CKD progression than standard office BP [105]. In individuals with CKD, there is a decrease in the production of endothelial NO, which plays a role in regulating BP. This reduction in NO synthesis may lead to elevated nighttime BP through two mechanisms. Firstly, it directly affects the endothelium, leading to impaired blood vessel function. Secondly, it indirectly influences nighttime BP by triggering heightened activity in the SNS [105,106]. In patients with CKD, several factors such as the increased nighttime excretion of salt, heightened activity in the SNS and RAAS, and sleep disturbances are common. These factors, whether occurring individually or together, disrupt the normal decrease in nighttime BP. Research indicates the link between masked hypertension and a lower estimated glomerular filtration rate (eGFR), which is more pronounced in patients with elevated nighttime BP. Interestingly, studies show that CKD patients who meet the nighttime BP target do not face a higher risk of kidney failure or cardiovascular events. Conversely, those who solely achieve the daytime BP target are at greater risk of cardiovascular issues [107]. Individuals with a riser or non-dipper pattern of nighttime BP often have increased circulating volume, primarily influenced by salt sensitivity and intake. This is supported by the fact that reducing salt intake and using diuretic medications effectively lower nighttime BP more than daytime BP, shifting the BP pattern from non-dipping to dipping during the day–night cycle [108]. When BP rises, the body’s response is to increase sodium excretion through pressure natriuresis. While individuals with sodium retention and sufficient circulating volume can manage with elevated daytime BP to eliminate excess sodium, those with increased circulating volume require elevated BP both during the day and at night to effectively excrete sodium. The supine position at night may also contribute to nocturnal hypertension in salt-sensitive individuals due to increased renal circulating volume while lying down, and possibly heightened sensitivity to plasma volume changes, leading to increased preload in the left ventricle. Additionally, being supine increases venous return to the heart, which further elevates preload and cardiac stress, triggering the release of natriuretic peptides due to stretching of the atria and ventricles [108,109,110]. Nocturnal hypertension is therefore one of the main risk factors for complications, mainly cardiovascular. The second major risk factor is resistant hypertension. Individuals with resistant hypertension are at a higher risk of experiencing damage to target organs, such as thickening of the carotid intima–media, left ventricular hypertrophy, impaired renal function, and microalbuminuria. Consequently, these patients face an unfavorable prognosis and are more prone to encountering adverse combined outcomes over time, including death, myocardial infarction, congestive heart failure, stroke, or CKD, compared to those who have attained their target BP [83].

## 4. Novel Biomarkers

It is well known that the currently widely used diagnostic markers are levels of serum creatinine (sCR) and blood urea. However, the increase in sCR levels occurs only when approximately 40–50% of renal parenchyma is reversibly or irreversibly damaged. Limitations in CKD diagnosis caused by the imperfections of sCr as a marker prompt researchers to seek biomarkers that are more sensitive than sCR [111].

Cystatin C is a protein with a molecular weight of 13 kDa, synthesized at a constant rate in all nucleated cells. Its biological function encompasses involvement in both physiological and pathological processes such as cell proliferation, cell migration, and cell apoptosis [112]. Cystatin C is detectable in nearly every tissue and organ of the body, with its concentration varying across body fluids. Notably, it reaches its highest concentration in semen, while being practically undetectable in urine [113,114]. The clearance of Cystatin C from the body predominantly occurs in the kidneys [115]. Scientific evidence strongly indicates that Cystatin C serves as a more sensitive marker of kidney damage compared to sCR levels. Importantly, serum levels of Cystatin C are not influenced by gender, age, or alcohol intake [116,117]. Furthermore, Cystatin C, as opposed to creatinine, allows for earlier detection of declining kidney function, and its serum concentration is less dependent on muscle mass [115]. In 2012, the Kidney Disease: Improving Global Outcomes (KDIGO) guidelines recommended the use of Cystatin C as a marker for CKD in cases where the early detection of CKD via sCR levels might be missed [118]. Cystatin C is a highly useful biomarker for estimating the GFR and assessing the progression of CKD, as well as cardiovascular risk [115]. It has been demonstrated that a combined creatinine–Cystatin C equation may serve as a valuable confirmatory test in CKD and is more accurate than equations based solely on either of these markers alone [119].

MCP-1 is a protein produced by kidney cells under pro-inflammatory conditions. The production of MCP-1 by tubular cells and podocytes increases in response to high serum glucose levels, and MCP-1 levels in urine are significantly higher in patients with DN than in the general population, as demonstrated in clinical research [120,121]. Additionally, MCP-1 levels in urine during the course of lupus nephropathy (LN) are also elevated compared to the general population.MCP-1 may prove to be a useful marker of CKD associated with chronic inflammation, although its concentration does not correlate with the degree of renal activity [122,123].

Connective Tissue Growth Factor (CTGF) belongs to the matricellular proteins, which participate in cell proliferation, adhesion, and wound-healing processes [124]. Kidney diseases associated with increased levels of CTGF include, for example, segmental and focal glomerulosclerosis, DN, and IgA nephropathy [125]. The concentration of CTGF correlates with the degree of renal function deterioration in DN and serves as an independent predictor of ESRD [126].

Fibroblast Growth Factor 23 (FGF-23) is a protein with a molecular weight of 32 kDa. It is primarily synthesized by osteoblasts and participates in the regulation of calcium-phosphate metabolism. Its biological functions include reducing the concentration of calcitriol, inhibiting the secretion of PTH, and inducing phosphaturia [127,128]. The concentration of FGF-23 increases with worsening kidney function, particularly when the GFR drops below 60 mL/min/1.73m^2^ [129]. Moreover, elevated levels of FGF-23 correlate with the rate of CKD progression and a higher risk of cardiovascular events [130,131].

Neutrophil Gelatinase-Associated Lipocalin (NGAL) is produced by tubular epithelial cells in response to tubulointerstitial damage and injury. Research has shown that NGAL serves as a valuable marker in the diagnosis of both CKD and AKI [132]. It has been demonstrated that serum levels of NGAL are higher in patients with CKD. Furthermore, NGAL levels positively correlate with the risk of cardiovascular events and CKD severity. Patients with elevated serum NGAL levels compared to those with low NGAL levels are more likely to experience deteriorating residual kidney function within one year [133].

Kidney Injury Molecule-1 (KIM-1) is a transmembrane glycoprotein found in the proximal tubule. In healthy kidneys, the concentration of this glycoprotein is undetectable, but in cases of CKD or AKI, both urine and serum levels significantly increase [133]. Elevated levels of KIM-1 have been observed in conditions such as DN, IgA nephropathy, hypertensive nephropathy, and LN [134]. A negative correlation between the GFR and urine KIM-1 levels has been demonstrated in IgA nephropathy and LN. In patients with DN, serum KIM-1 levels correlate with GFR reduction, suggesting that this glycoprotein may serve as an early marker of CKD [135]. However, studies conducted in 2018 did not show a significant correlation between urine KIM-1 levels and CKD [136].

In recent years, new biomarkers such as NGAL, KIM-1, Beta trace protein (BTP), and the previously discussed ADMA have been employed to identify the progression of CKD. In a study conducted in 2023, the concentrations of these biomarkers were compared between 100 CKD patients and 100 healthy individuals. It was demonstrated that the concentrations of biomarkers such as ADMA, KIM-1, NGAL, and BNP were significantly higher in CKD patients compared to healthy individuals. Moreover, a positive correlation was found between the concentrations of these biomarkers and the serum levels of creatinine and urea, while a negative correlation was observed between the concentrations of these biomarkers and creatinine clearance. Based on these findings, it can be inferred that regular monitoring of the concentrations of these biomarkers may be helpful in the early detection of CKD [137].

In a study conducted in 2022, it was demonstrated that lower levels of biomarkers such as fibrinogen, fractalkine, brain natriuretic peptide (BNP), high-sensitivity troponin-T (hsTnT), BTP, and KIM-1 are associated with a slower deterioration of kidney function among patients with non-albuminuric CKD in DN compared to those patients with normal kidney function [138].

## 5. Conclusions

The kidneys are responsible for a variety of mechanisms involved in maintaining the body’s homeostasis. They regulate the acid–base, ion, and water balance. Additionally, they control BP, eliminate unnecessary metabolic waste products from the bloodstream, and secrete selected hormones and enzymes. Unfortunately, the number of patients suffering from renal dysfunction in the form of CKD is increasing year by year. Various factors contribute to the development of CKD, but particular attention should be paid to uremic toxins, VC, TGF-β1, and RAAS activity. It is important to remember that CKD is largely associated with a chronic pathological increase in activation of the RAAS, particularly angiotensin II and aldosterone. This contributes to the acidosis of renal tubules, their atrophy, mitochondrial dysfunction, increased levels of oxidative stress, and chronic inflammation. It is also important to be aware of the cardio-renal syndrome. Pathological RAAS stimulation through heart failure secondarily causes CKD. Hypertension and CKD share a complex interplay, where high BP exacerbates kidney function decline, and deteriorating kidney function further complicates BP control. Effective management of hypertension in CKD involves regular BP monitoring and lifestyle modifications including diet, exercise, and pharmacological interventions. Resistant hypertension in CKD patients increases the risk of target organ damage and adverse cardiovascular outcomes, underscoring the importance of comprehensive management strategies to mitigate complications. Also, TGF-β1 plays a valid role in the initiation and development of renal fibrosis, which is crucial in CKD, but the precise mechanism is still not well understood. Moreover, CKD patients more frequently present VC, which is caused by mineral compound metabolism dysfunctions. In recent years, numerous novel biomarkers have emerged that may serve as indicators of deteriorating kidney function or as more sensitive diagnostic markers for CKD, with Cystatin C appearing to be the most promising. Despite the abundance of these molecules and their wide-ranging applications in CKD, we require more scientific evidence to identify a reliable and universal biomarker. The impact of uremic toxins on the progression of CKD has been extensively investigated in recent years. A more comprehensive understanding of the properties of these substances could facilitate the development of methodologies for their removal from the organism and contribute to strategies aimed at mitigating the progression of the disease.

## Figures and Tables

**Table 1:** Categories of chronic albuminuria based on albumin–creatinine ratio in urine [7].

Chronic Albuminuria Category	Albumin–Creatinine Ratio in Urine
A1	<3 mg/mmol <30 mg/g
A2	3–30 mg/mmol 30–300 mg/g
A3	>30 mg/mmol >300 mg/g

**Table 2:** CKD categories based on GFR [7].

GFR Category	GFR (mL/min/1.73 m^2^)
G1	≥90
G2	60–89
G3a	45–59
G3b	30–44
G4	15–29
G5	<15

**Table 3:** Impact of different molecules on chronic kidney disease [38,40,58,63,69,70,78].

Molecules	Impact on Chronic Kidney Disease
TGF-β	Induceskidney fibrosis
p53	Development of acute kidney injury
IS	Induceskidney inflammation and fibrosis, nephrotoxic effect
pCS	Induceskidney inflammation and fibrosis
TNF-α	Leads to reduction in GFR
TNFR2	Glomerulosis and interstitial renal fibrosis
TMAO	Accumulation leads to tubulointerstitial renal fibrosis

TGF-β—transforming growth factor-β; IS—indoxyl sulfate; pCS—p-cresyl sulfate; TNF-α—tumor necrosis factorα; TNFR2—tumor necrosis factor receptor type 2; TMAO—trimethylamine N-oxide.

## Data Availability

The data used in this article were sourced from the materials mentioned in the References section.
